# WNT5B governs the phenotype of basal-like breast cancer by activating WNT signaling

**DOI:** 10.1186/s12964-019-0419-2

**Published:** 2019-08-28

**Authors:** Shaojie Jiang, Miaofeng Zhang, Yanhua Zhang, Weiping Zhou, Tao Zhu, Qing Ruan, Hui Chen, Jie Fang, Fei Zhou, Jihong Sun, Xiaoming Yang

**Affiliations:** 1 Department of Radiology, Sir Run Run Shaw Hospital, School of Medicine, Zhejiang University, Hangzhou, 310016 Zhejiang China; 2 Department of Orthopaedics, Second Affiliated Hospital, School of Medicine, Zhejiang University, Hangzhou, 310009 Zhejiang China; 3 Department of Pathology, Sir Run Run Shaw Hospital, School of Medicine, Zhejiang University, Hangzhou, 310016 Zhejiang China; 4 Department of Diagnostic Ultrasound and Echocardiography, Sir Run Run Shaw Hospital, School of Medicine, Zhejiang University, Hangzhou, 310016 Zhejiang China; 5 Department of Surgery, Division of Hepatobiliary and Pancreatic Surgery, The First Affiliated Hospital, School of Medicine, Zhejiang University, Hangzhou, 310003 Zhejiang China; 6 Key Laboratory of Experimental Animal and Safety Research, Zhejiang Academy of Medical Sciences, Hangzhou, 310013 Zhejiang China; 7 Image-Guided Bio-Molecular Intervention Research, Department of Radiology, University of Washington School of Medicine, Seattle, Washington 98109 USA

**Keywords:** WNT5B, Basal-like breast cancer, Luminal breast cancer, Canonical/non-canonical Wnt signaling, Epithelial-mesenchymal transition

## Abstract

**Background:**

Breast cancer is the leading cause of cancer-related death in women worldwide. Metastatic disease remains the primary cause of death in patients with breast cancer. Basal-like breast cancer (BLBC) is associated with aggressive behavior, stem-like phenotype, high histological grade, poor clinical features, and high rates of recurrences and/or metastasis. However, the mechanism of BLBC phenotype shaping remains obscure.

**Methods:**

Seventeen normal breast/breast cancer cell lines were used for evaluating the breast cancer subtype-markers, WNT targets and constitutive components, and epithelial mesenchymal transition (EMT) markers analysis by western blot. One hundred and twenty formalin-fixed breast cancer tissues were used for immunohistochemistry (IHC) staining. Nine online platforms (cBioPortal, CCLE, GEPIA, etc.) were used for related analyses.

**Results:**

We identified Wnt5b as a key regulatory factor that governs the phenotype of BLBC by activating canonical and non-canonical WNT signaling. Wnt5b exhibited basal-like specificity in cells and clinical samples both at the mRNA and protein levels and also showed good correlation with basal-like phenotype at the mRNA level. Besides, Wnt5b was also a promising therapeutic target for LGK-974 treatment. In addition, we identified that CK1α was expressed at low levels in BLBC and that the activation of CK1α by pyrvinium was an alternative strategy for BLBC treatment.

**Conclusions:**

Wnt5b is not only a diagnostic biomarker but also a potential therapeutic target of BLBC.

**Graphical abstract:**

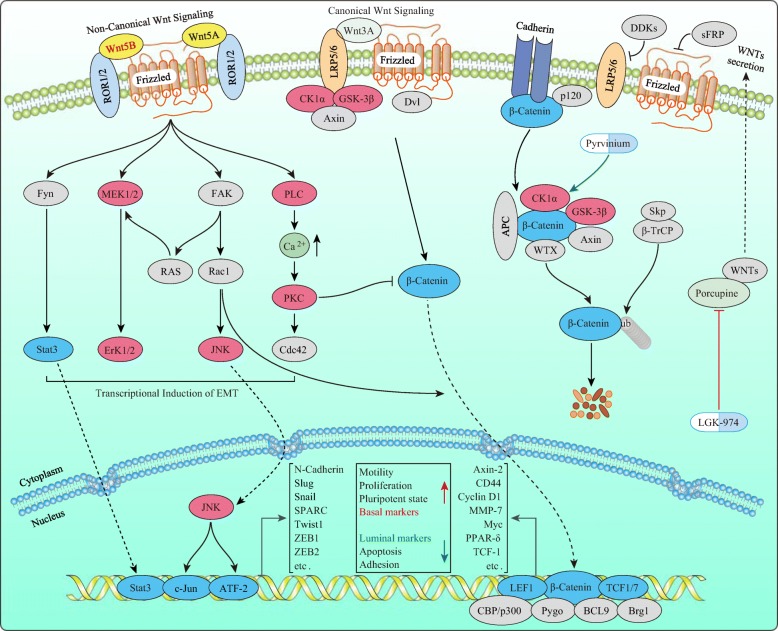

**Electronic supplementary material:**

The online version of this article (10.1186/s12964-019-0419-2) contains supplementary material, which is available to authorized users.

## Background

Breast cancer is the most commonly diagnosed cancer (24.2% of the total cases) and the leading cause of cancer death (15% of the total cancer deaths) among females worldwide in 2018 [1]. Classification of breast cancer into luminal A, luminal B, Her-2+, and basal-like subtypes based on gene expression profiles has significantly changed the understanding and treatment of breast cancer [2–4]. Later, Basal-like breast cancer (BLBC) was defined as estrogen receptor (ER) negative, human epidermal growth factor receptor 2 (Her-2) negative; epidermal growth factor receptor (EGFR, also known as Her-1) or/and Cytokeratin 5/6 (CK 5/6) positive [5].

A previous study reported 51% luminal A, 16% luminal B, 7% Her-2+, 20% basal-like, and 6% normal-like breast cancers in 496 cases of invasive breast cancer [6]. Thus, BLBC is the second most common subtype after luminal A of invasive breast cancer. BLBC is often associated with metastatic disease and the incidence of metastasis in the BLBC is second to the Her-2-enriched subtype. Notably, BLBC has the shortest median survival (0.5 years) among all subtypes with distant metastasis [7]. Thus, BLBC is the most fatal subtype among breast cancer subtypes. Previous studies demonstrated that BLBC cell lines expressed EMT-acquired markers such as Snail, vimentin, and N-cadherin, but lost EMT-attenuated marker E-cadherin compared to luminal breast cancer cell lines [8, 9]. Various studies have indicated that both the canonical and non-canonical Wnt signaling is involved in the metastasis of breast cancer via the EMT pathway [9–12]. However, whether canonical or non-canonical Wnt signaling contributes to the metastasis of all breast cancers subtypes is relatively unclear.

Canonical Wnt signaling is associated with the metastasis of various cancers, including breast cancer [13]. Wnt ligands and Wnt constructive components are frequently mutated, and over- or under-expressed in many different human cancers – in particular, adenomatous polyposis coli (*APC*) in colorectal cancer [14], *CTNNB1* (encoding β-Catenin) in colon adenocarcinoma [15], lung adenocarcinoma [16], and endometrial carcinoma [17], and *WTX* (Wilms tumor gene on the X chromosome, also known as *FAM123B*) in Wilms tumor [18], etc. are the most commonly dysregulated canonical Wnt elements. However, no or very few *CTNNB1* mutations have been reported in breast cancer [19]. In addition, the subcellular localization of β-Catenin differs in breast cancer subtypes – invasive ductal carcinomas exhibited membranous β-Catenin staining, BLBC exhibited strong nuclear β-Catenin staining, while lobular carcinomas lacked β-Catenin expression [13, 19].

Non-canonical Wnt signaling is grouped into several categories including Wnt/planar cell polarity (PCP), Wnt-cGMP/Ca^2+^, Wnt-RAP1, and Wnt-ROR2 (Receptor tyrosine kinase-like orphan receptor 2). All these types of non-canonical Wnt signaling are characterized as Wnt- or Frizzled (Fzd)-initiated but β-Catenin independent [20]. Wnt5a is a non-canonical Wnt ligand which is overexpressed specifically in BLBC cell lines, and the inhibition of the Twist-bromodomain containing 4 (BRD4) association by JQ1 reduced Wnt5a expression and suppressed invasion, cancer stem cell (CSC)-like property, and tumorigenicity of BLBC cells [21]. Fibroblast-secreted exosomes promote the protrusive activity and motility of breast cancer cells through Wnt-PCP signaling [10]. Non-canonical Wnt receptor Fzd2 and its ligands Wnt5a/b are elevated in metastatic breast cancer cell lines and in high-grade tumors and that their expression correlates with markers of EMT [11].

However, the mechanisms by which the BLBC cells maintain their physical and physiological phenotype remain obscure. In this study, we examined the factors affecting the physical and physiological phenotype of BLBC cells and identified WNT5B as a key factor required for shaping the phenotype of BLBC cells.

## Methods

### Cell lines

All breast cell lines were purchased from the CBCAS (Cell Bank of the Chinese Academic of Sciences, Shanghai, China). All cell lines except Bcap-37 were maintained in culture as described in a previous report [22]. Bcap-37, a Chinese breast cancer cell line established by Changwei Chen (J. Beijing Medical College, 1983, 15(3): 161), was maintained in RPMI 1640 medium (Gibco, 11,875–085) with 10% fetal bovine serum (Gibco, 1099–141). The cell lines were divided into the normal group, luminal group, and basal-like group based on a previous study [22].

### Western blot analysis

All cell lines were harvested at ~ 80% confluence and then lysed using RIPA buffer (Thermo Scientific, #89901). Protein concentration was measured using the BCA protein assay kit (Thermo Scientific, #23225). Protein samples were resolved by 8~12% SDS-PAGE and transferred to a PVDF membrane (Bio-Rad, #162–0117). The membrane was blocked in TBS containing 0.05% Tween-20 (Amresco, 0777-1 L) with 5% non-fat skim milk (BD, #232100) for 1 h at 25 °C, followed by overnight incubation with primary antibodies overnight at 4 °C. After three washes in TBST, the membrane was incubated with horseradish peroxidase (HRP)-conjugated secondary antibody for 1 h at room temperature. After three washes in TBST, the membrane was added the membrane was treated with the EZ-ECL kit (Biological Industries, #20–500-120) reagent, and visualized using a Tanon-5200 multi-automatic chemiluminescence/fluorescence imaging analysis system (Tanon Science & Technology Inc., Shanghai, China).

### Antibodies for western blot

Canonical breast cancer subtypes markers: anti-Her-2/ErbB2 (CST, #2165), anti-Progesterone Receptor A/B (CST, #8757), anti-Estrogen Receptor α (CST, #8644), anti-Keratin 18 (CST, #4548), anti-FoxA1/HNF3α (CST, #58613), anti-AGR2 (CST, #13062), anti-CD44 (CST, #3570), anti-Caveolin-1 (CST, #3267), anti-Caveolin-2 (CST, #8522), anti-EGF Receptor (CST, #4267), anti-Cytokeratin 5 (Sigma, SAB5300267), and anti-SPARC (CST, #5420).

Wnt signaling targets: anti-Met (CST, #8198), anti-CD44 (CST, #3570), anti-TCF1/7 (CST, #2203), anti-c-Jun (CST, #9165), anti-LEF1 (CST, #2230), anti-c-Myc (CST, #5605), anti-Cyclin D1 (CST, #2978), anti-MMP-7 (Abcam, ab207299), and anti-Axin2 (CST, #2151).

Canonical Wnt signaling constitutive components: anti-Phospho-LRP6 (Ser1490) (CST, #2568), anti-LRP6 (CST, #3395), anti-Dvl2 (CST, #3224), anti-Dvl3 (CST, #3218), anti-APC (CST, #2504), anti-Axin1 (CST, #2087), anti-CK1α (Santa cruz, sc-6477), anti-GSK-3α/β (CST, #5676), anti-Non-phospho (Active) β-Catenin (Ser45) (CST, #19807), anti-Non-phospho (Active) β-Catenin (Ser33/37/Thr41) (CST, #8814), anti-Phospho-β-Catenin (Ser552) (CST, #5651), anti-Phospho-β-Catenin (Ser675) (CST, #4176), anti-Phospho-β-Catenin (Thr41/Ser45) (CST, #9565), anti-Phospho-β-Catenin (Ser33/37/Thr41) (CST, #9561), anti-β-Catenin (CST, #8480).

EMT markers: anti-E-cadherin (CST, #3195), anti-ZO-1 (CST, #8193), anti-N-cadherin (CST, #13116), anti-Claudin-1 (CST, #13255), anti-β-Catenin (CST, #8480), anti-Vimentin (CST, #5741), anti-TCF8/ZEB1 (CST, #3396), anti-Snail (CST, #3879), anti-Slug (CST, #9585), and anti-TWIST1 (CST, #46702).

Non-canonical Wnt signaling components: anti-Phospho-JNK (CST, #9251), anti-JNK (CST, #9252), anti-Phospho-Stat3 (Tyr705) (CST, #9145), anti-Stat3 (CST, #12640), and anti-Erk1/2 (CST, #9102).

Other antibodies: anti-Wnt3a (Abcam, ab81614), anti-Wnt5a (HUABIO, #ET1706–33), anti-Wnt5b (Abcam, ab124818). anti-β-Actin (CST, #3700), secondary antibodies including anti-rabbit IgG, HRP-linked Antibody (CST, #7074), and anti-mouse IgG, HRP-linked Antibody (CST, #7076). All primary antibodies that used for western blot were diluted into 1: 1000, and secondary antibodies that used for western blot were diluted into 1: 2000.

### Clinical specimens, IHC, and immunofluorescence (IF) staining

Formalin-fixed and paraffin-embedded primary breast cancer tissues were obtained from Sir Run Run Shaw Hospital, School of Medicine, Zhejiang University. Molecular subtypes were histologically characterized by three pathologists. All human tissues were obtained with written informed consents and with the approval of the Medical Ethical Committee of Sir Run Run Shaw Hospital. IHC staining was performed following our previously reported protocol [23]. The following antibodies were used for IHC staining – anti-Her-2/ErbB2 (Roche, 790–4493), anti-PR (Novocastra, NCL-L-PGR-312), anti-ER (Novocastra, NCL-L-ER-6F111), anti-Ki-67 (Dako, M7240), anti-EGFR (Roche, 790–4347), anti-CK-5/6 (Dako, M7237), anti-Wnt5b (Abcam, ab94914), and REAL™ EnVision™ Detection System, HRP/DAB, Rabbit/Mouse kit (Dako, K5007). The other antibodies were the same as those used for western blot. Anti-β-Catenin (CST, #8480), Anti-rabbit IgG (H + L), F (ab′)2 Fragment (Alexa Fluor® 555 Conjugate) (CST, #4413), DyLight™ 554 Phalloidin (CST, #13054), and DAPI Staining Solution (Beyotime, C1006) were used for IF staining, which was performed according to the protocol from (https://www.cellsignal.com/). Breast cancer cells were treated with or without recombinant human Wnt3a (200 ng/mL, R&D, 5036-WN/CF), Wnt5a (500 ng/mL, R&D, 645-WN/CF), or Wnt5b (500 ng/mL, R&D, 7347-WN/CF) protein for 24 h before IF staining. All primary antibodies that used for IHC/IF were diluted into 1: 200, and secondary antibodies that used for IHC/IF were diluted into 1: 2000.

### Bioinformatic analysis based on online platforms

mRNA expression of the indicated genes among basal-like, Her-2+, luminal A, luminal B, and normal-like breast cancer patients according to Sørlie’s subtypes [3, 4] was assessed by Breast Cancer Gene-Expression Miner v4.1 (bc-GenExMiner v4.1) (http://bcgenex.centregauducheau.fr) [24, 25]. The *P* value was also calculated by bc-GenExMiner v4.1. mRNA expression of the indicated genes among normal, Her-2+, luminal and triple negative breast cancer (TNBC) patients were assessed by UALCAN (http://ualcan.path.uab.edu/) [26]. The *P* value was also calculated by UALCAN.

A total of 6370 sequenced cases/patients from 11 invasive breast carcinoma studies were selected for amplification (AMP) or copy-number alteration (CNA) analyses from the cBio Cancer Genomics Portal (cBioPortal) (http://www.cbioportal.org/) [27, 28]. The survival analysis between the *Myc* or/and *CCND1* AMP group and the *Myc*/*CCND1* normal group was also obtained from the cBioPortal.

WNT3A, WNT5A, and WNT5B mRNA expression levels across various normal tissues were derived from the Genotype-Tissue Expression (GTEx) (https://gtexportal.org/) [29] which deposited in the Human Protein Atlas (HPA) (https://www.proteinatlas.org/) [30].

Breast cancer samples from patients were divided into WNT3, WNT5A, WNT5B, WNT11-high and WNT3, WNT5A, WNT5B, WNT11-low-expression groups by auto-cutoff according to the mRNA expression value, and the prognosis of overall survival (OS) and relapse free survival (RFS) were assessed by Kaplan-Meier Plotter (KM-plotter) (http://kmplot.com/) [31]. The *P* value was also calculated by KM-plotter.

mRNA expression data of the indicated genes in heatmaps were extracted from the Cancer Cell Line Encyclopedia (CCLE) (https://portals.broadinstitute.org/ccle) [32] and displayed using GraphPad Prism 7 software. Thirty-four breast cancer lines were divided into a luminal group and a basal-like group according to the previous study [22].

Correlation between WNT5B or CSNK1A1 and other indicated genes according to the mRNA expression value was calculated by GEPIA (http://gepia.cancer-pku.cn/) [33]. The *P* value and *R*-value were also calculated by GEPIA.

Protein-Protein interaction (PPI) pattern of WNT3A, WNT5A, and WNT5B was based on (STRING) (https://string-db.org/) [34].

### Semi-quantitative reverse transcription-polymerase chain reaction (PCR) analysis

Total cellular RNA was extracted from two normal breast cell lines and 15 breast cancer cell lines (about 80% confluence) using RNeasy® plus micro kit (QIAGEN, 74034). The RNA concentration was measured using a NanoDrop 2000 (Thermo Fisher Scientific, ND2000). Subsequent semi-RT-PCR was performed using the AccessQuick™ RT-PCR System (Promega, A1702). Semi-RT-PCR products were analyzed by electrophoresis on a 1% agarose gel. Primer sequences (F, forward; R, reverse; 5′➔3′) are listed below.

- *WNT3A*-F: GTGTTCCACTGGTGCTGCTA,
- *WNT3A*-R: AGAGGAGACACTAGCTCCAGG;
- *WNT5A*-F: TGCAAGAAGTGCACGGAGAT,
- *WNT5A*-R: TTCCCACCCCCATTATTGCC;
- *WNT5B*-F: AGAGGCCTGGTGCTCTCTTA,
- *WNT5B*-R: AGTATAACGTCCACGCAGCC;
- *GAPDH*-F: GAAGGTGAAGGTCGGAGTC,
- *GAPDH*-R: AAGATGGTGATGGGATTTC.

### Virus production and transfection

shRNAs targeting *WNT5B* were cloned into the pLent-U6-RFP-Puro (Vigne, #LT88024) vector. Targeting sequences were as follows:

- shRNA-Ctr: 5′-GCACCCAGUCCGCCCUGAGCAAA-3′,
- shWNT5B-1: 5′-GGAAAGGAAGAGCUUAUUUAA-3′,
- shWNT5B-2: 5′-GUGGACCAGUACAUCUGUAAA-3′,
- shWNT5B-3: 5′-GAAUUGCAGCACAGCGGACAA-3′.

The 293 T cell line was used for lentivirus packaging. Briefly, a 10-cm dish of 4 × 10^6^ non-confluent 293 T cells was co-transfected with recombinant pLent-U6-RFP-Puro, pMD2G (Addgene, #12259), and psPAX2 (Addgene, #12260). The lentivirus-containing supernatant was harvested after 48 h and used for the subsequent experiment.

### Transwell assay

A 24-well transwell chamber (Costar, 3422) precoated with or without Matrigel (BD, 354248; dilution at 1:8) was used to detect cell migration or invasion according to the manufacturer’s protocol. MDA-MB-231 and Bcap-27 with WNT5B-knockdown (KD); or sh-control with or without Wnt5b (500 ng/mL) suspended in 0.2 mL serum-free medium (1 × 10^5^ Bcap-37 cells/well for migration, 1.5 × 10^5^ Bcap-37 cells/well for invasion; 2 × 10^5^ MDA-MB-231 cells/well for migration, 3 × 10^5^ MDA-MB-231 cells/well for invasion) were added to the upper chambers, and medium supplemented with 10% FBS was applied to the bottom chambers. After incubating the cells for 24 h (for migration) or 48 h (for invasion), the cells that migrated to the lower membrane surface were fixed and stained with crystal violet solution.

### Foci formation assay

Foci formation assay was performed as described previously [35]. Briefly, 5000 cells were seeded in six-well culture plates in 2 mL growth media. After 12 h the media was replaced with fresh growth media containing 1 μM LGK974 or 200 nM pyrvinium pamoate in the absence or presence of recombinant Wnt5b (500 ng/mL). When cell colonies reached a desirable size, cells were fixed with 10% (vol/vol) formalin in phosphate-buffered saline (PBS) and stained with crystal violet solution. After three washes, plates were dried and imaged.

### Cell viability

Cell viability was performed by using CellTiter-Glo® Luminescent Cell Viability Assay (Promega, G7570) according to its protocol & application guide.

### In vivo tumorigenicity, fluorescence and ultrasound imaging

Breast cancer cell lines MDA-MB-231 and Bcap-37 were used for xenograft transplantation in BALB/c nude mice as described previously [36]. Briefly, 5 × 10^6^ Bcap-37 cells in 100 μL serum-free PBS, or 5 × 10^6^ MDA-MB-231 cells (were resuspended in a 1:1 mixture of 100 μL PBS and Matrigel [BD, 354248; dilution at 1:4]) were injected subcutaneously into each flank of locally bred BALB/c-nu mice (5 weeks old, female; 4~5 mice per group). The tumor size was measured every 3 days for Bcap-37 and 5 days for MDA-MB-231, and tumor volume was calculated using the formula (length×width×width)/2. When tumors reached a 1000 mm^3^ size the mice were sacrificed. Images of breast cancer cell-bearing mice were acquired using a Clairvivo OPT plus imaging system (SHIMADZU). Briefly, mice were anesthetized using 2% isoflurane for 5 min using the RC2 Anesthesia Machine (VetEquip). The mice were then imaged under 530 nm laser irradiation. Ultrasound imaging was performed following our previously reported protocol [23].

### Statistical analyses

All data were analyzed using GraphPad Prism 7 software and are represented as the mean ± standard error of the mean (SEM) unless otherwise indicated. Statistical analyses were performed using an unpaired Student’s *t*-test. For the tumor growth curve, data were assessed by two-way ANOVA method, and *P* < 0.05 was considered to indicate statistical significance. For the IHC data, data were assessed by Pearson’s χ^2^ test. For all comparisons, *P* < 0.05 was considered to indicate statistical significance.

## Results

### Analyses of biomarkers of breast cancer subtypes in normal breast/breast cancer cell lines and clinical samples

**Fig. 1:**
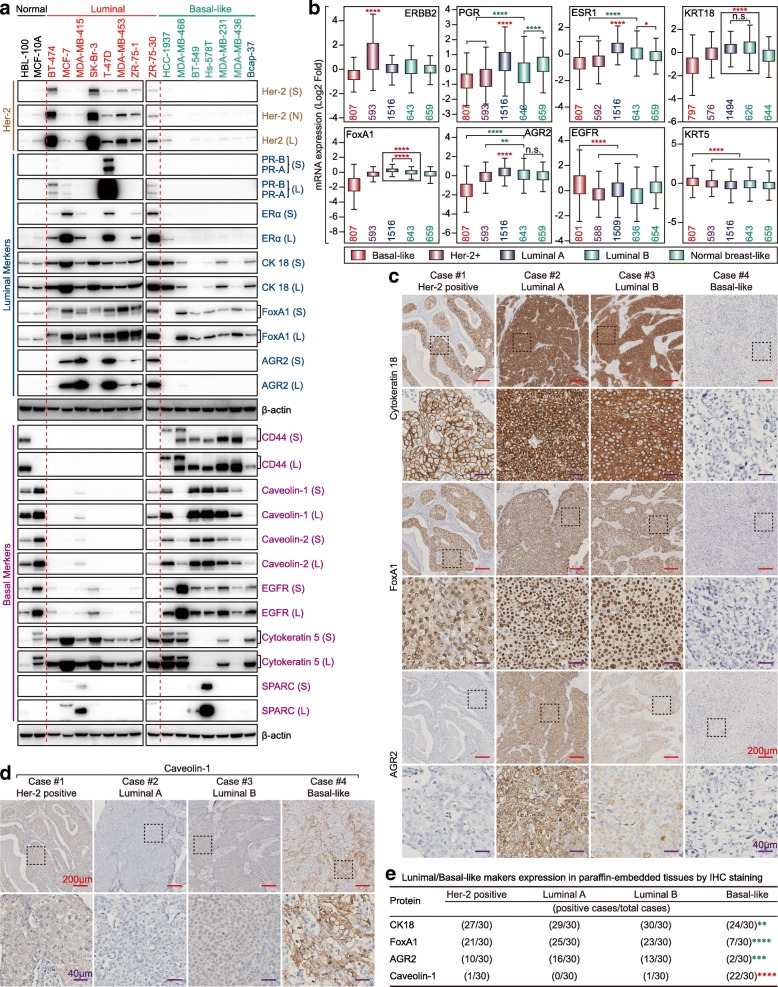
Expression of canonical breast cancer subtype-markers in breast cancer cells and clinical samples. **a** Expression of canonical breast cancer subtype-markers in two normal breast cell lines, eight luminal, and seven BLBC cell lines by western blot (S: Short exposure; L: Long exposure). **b** Expression levels of selected canonical breast cancer subtype-marker mRNAs with Her-2 positive, luminal or basal-like specific based on bc-GenExMiner v4.1 according to the Sørlie’s subtypes (*p < 0.05; ****p < 0.0001; Red star: the former > the latter; Green Star: the former < the latter). Expression of selected luminal breast cancer markers (**c**) and basal-like specific marker Caveolin-1 (**d**) in representative Her-2 positive, luminal A, luminal B and basal-like breast cancer tissues by IHC (Red scale bar = 200 μm; Purple scale bar = 40 μm). **e** The association of selected luminal or basal-like specific markers expression between basal-like and non-BLBC tissue subtypes were assessed using Pearson’s χ2 test (for CK18, *p* = 0.0076; for FoxA1, *p* < 0.0001; for AGR2, *p* = 0.0002; for Caveolin-1, *p* < 0.0001; Red star, basal-like > non-basal-like; Green star, basal-like < non-basal-like)

We collected two normal breast cell lines, eight luminal, and seven BLBC cell lines. Her-2 as a marker for Her-2+ breast cancer; PR, ER, CK18 (Cytokeratin 18), FoxA1 (also called HNF3α), and AGR2 (Anterior gradient 2) as markers of luminal breast cancer; CD44, Caveolin-1, Caveolin-2, EGFR, CK5 (Cytokeratin 5), and SPARC (Secreted protein acidic and cysteine-rich) as markers of BLBC were used for molecular typing. Most markers exhibited specificity, except CK5 and SPARC (Fig. 1a). Importantly, Bcap-37, a Chinese breast cancer cell line was identified as a BLBC cell line for the first time. However, Caveolin-1, Caveolin-2, and SPARC did not exhibit specificity at the mRNA level for BLBC (Fig. 1b) and TNBC (Additional file 1: Figure S1a) (Data of non-differential genes can be found in bc-GenExMiner and UALCAN. For CD44, also acts as a Wnt target, see Fig. 3e and Additional file 1: Figure S2a). In addition, we collected breast cancer samples of four subtypes (30 samples/group). IHC staining showed that CK18, FoxA1, and AGR2 exhibited specificity for the luminal subtype, while Caveolin-1 exhibited specificity for the BLBC (Fig. 1c-e) in addition to the conventional makers, Her-2, ER, PR, Ki-67, EGFR, and CK-5/6 (Additional file 1: Figure S1b). Thus, these markers are useful for molecular typing; further, analyzing the protein level may be more accurate than mRNA level while classifying breast cancer subtypes.

### Analyses of EMT biomarkers in normal breast/breast cancer cell lines and clinical samples

**Fig. 2:**
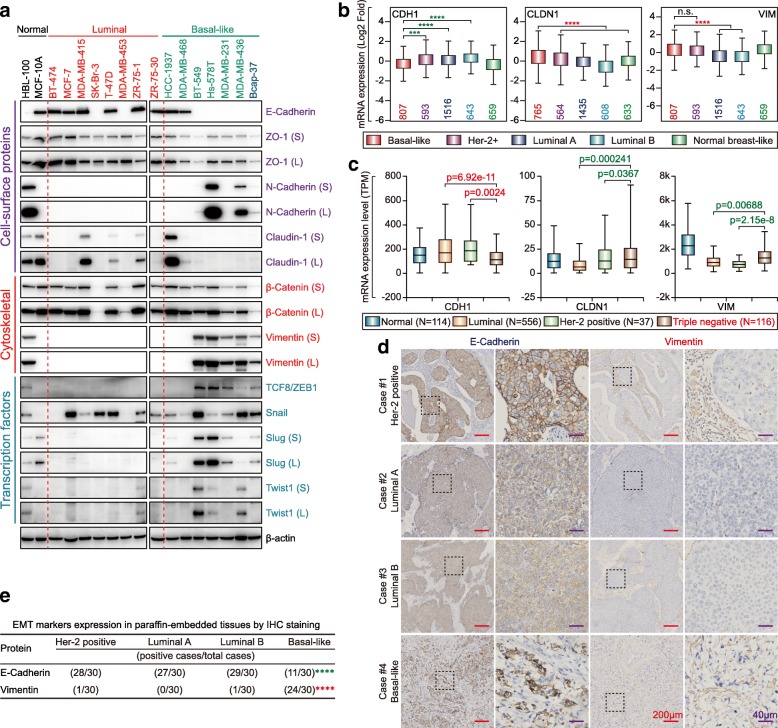
Expression of EMT markers in breast cancer cells and clinical samples. **a** Expression of EMT markers in two normal breast cell lines, eight luminal, and seven BLBC cell lines by western blot (S: Short exposure; L: Long exposure). **b** Expression of selected EMT marker mRNAs in basal-like specific based on bc-GenExMiner v4.1 according to the Sørlie’s subtypes (***p < 0.001; ****p < 0.0001; Red star: the former > the latter; Green Star: the former < the latter). **c** Expression of selected EMT marker mRNAs in TNBC-specific based on UALCAN (Red p: the former > the latter; Green p: the former < the latter). **d** Expression of E-cadherin and vimentin in representative Her-2 positive, luminal A, luminal B and basal-like breast cancer tissues by IHC (Red scale bar = 200 μm; Purple scale bar = 40 μm). **e** The association of E-cadherin or vimentin expression between basal-like and non-BLBC tissue subtypes were assessed using Pearson’s χ^2^ test (for E-cadherin, *p* < 0.0001, basal-like < non-basal-like; for vimentin, *p* < 0.0001, basal-like > non-basal-like)

Loss of attenuated EMT markers but overexpression of acquired EMT markers is another property of BLBC [8, 9]. E-cadherin, ZO-1, N-cadherin, Claudin-1, β-Catenin, vimentin, ZEB1, Snail, Slug, and Twist1 were selected as EMT markers based on a previous review [37]. As expected, E-cadherin, an attenuated EMT marker was lost in most of the BLBC cell lines, while most acquired EMT markers were preferentially expressed in BLBC cell lines, especially N-cadherin, vimentin, ZEB1, Slug, and Twist1 (Fig. 2a). CDH1 (encoding E-cadherin) was expressed at a low level, while CLDN1 (encoding Claudin-1) and VIM (encoding vimentin) were highly expressed in BLBC (Fig. 2b) and TNBC (Fig. 2c). We next examined the expression of E-cadherin and vimentin in breast cancer clinical samples and observed similar results (Fig. 2d and e).

### Most canonical Wnt signaling targets are preferentially overexpressed in BLBC

**Fig. 3:**
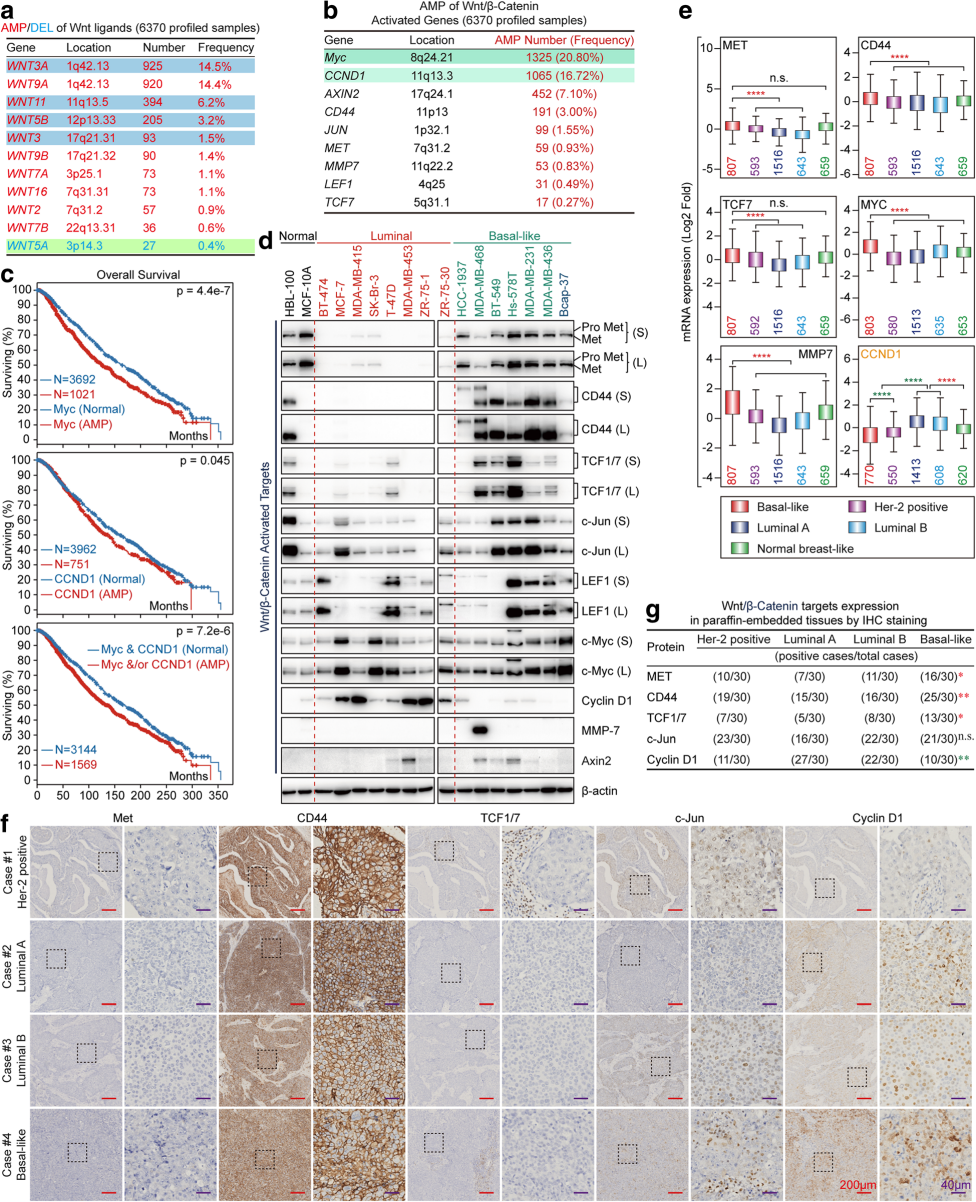
Canonical WNT signaling targets are preferentially overexpressed in BLBC. **a** List of Wnt ligand genes with AMP/DEL in 6370 invasive breast cancer patients based on TCGA data (AMP: Amplification; DEL: deletion). **b** List of Wnt/β-Catenin target genes with AMP in 6370 invasive breast cancer patients based on TCGA data (AMP: Amplification). **c** Overall survival analysis between *Myc* normal group and *Myc* amplified group (left); between *CCND1* normal group and *CCND1* amplified group (middle); and between *Myc* & *CCND1* normal group and *Myc* &/or *CCND1* amplified group (right). **d** Expression of canonical WNT signaling targets in two normal breast cell lines, eight luminal, and seven BLBC cell lines by western blot (S: Short exposure; L: Long exposure). **e** Expression levels of selected canonical WNT signaling target mRNAs with luminal or basal-like specific based on bc-GenExMiner v4.1 according to the Sørlie’s subtypes (****p < 0.0001; Red star: the former > the latter; Green Star: the former < the latter). **f** Expression of canonical WNT signaling targets in representative Her-2 positive, luminal A, luminal B and BLBC tissues by IHC (Red scale bar = 200 μm; Purple scale bar = 40 μm). **g** The association of canonical WNT signaling targets expression between basal-like and non-BLBC tissue subtypes were assessed using Pearson’s χ^2^ test (for MET, *p* = 0.0287; for CD44, *p* = 0.0065; for TCF1/7, *p* = 0.0249; for c-Jun, *p* = 0.6547; for Cyclin D1, *p* = 0.0013; Red star, basal-like > non-basal-like; Green star, basal-like < non-basal-like; n.s.: no significant)

A total of 6370 sequenced cases/patients from 11 invasive breast carcinoma studies were selected for AMP analysis based on cBioPortal. Wnt ligands, such as *WNT3A*, WNT9A, WNT11 and WNT5B were highly amplified in breast cancers (Fig. 3a). Further, *Myc* (encoding c-Myc) and *CCND1* (encoding Cyclin D1), *AXIN2*, etc. as canonical Wnt signaling targets were highly amplified in breast cancers (Fig. 3b). Moreover, breast cancer with *Myc* and/or *CCND1* exhibited poorer overall prognosis (Fig. 3c). So, we examined the expression of c-Myc and Cyclin D1, as well as other Wnt targets in the indicated cell lines. We found that most canonical Wnt signaling targets, especially Met, CD44, and TCF1/7 were preferentially overexpressed in BLBC cell lines. Surprisingly, Cyclin D1 was preferentially overexpressed in luminal breast cancer cell lines (Fig. 3d). Similarly, Met, CD44, TCF1/7, Myc, and MMP-7 were preferentially overexpressed in BLBC and TNBC, while Cyclin D1 showed luminal-specificity at the mRNA level (Fig. 3e and Additional file 1: Figure S2a). Furthermore, Met, CD44, and TCF1/7 were also preferentially expressed in BLBC clinical samples while Cyclin D1 was preferentially expressed in luminal breast cancer clinical samples (Fig. 3f and g).

### WNT5B is a specific marker of BLBC with poor prognosis

**Fig. 4:**
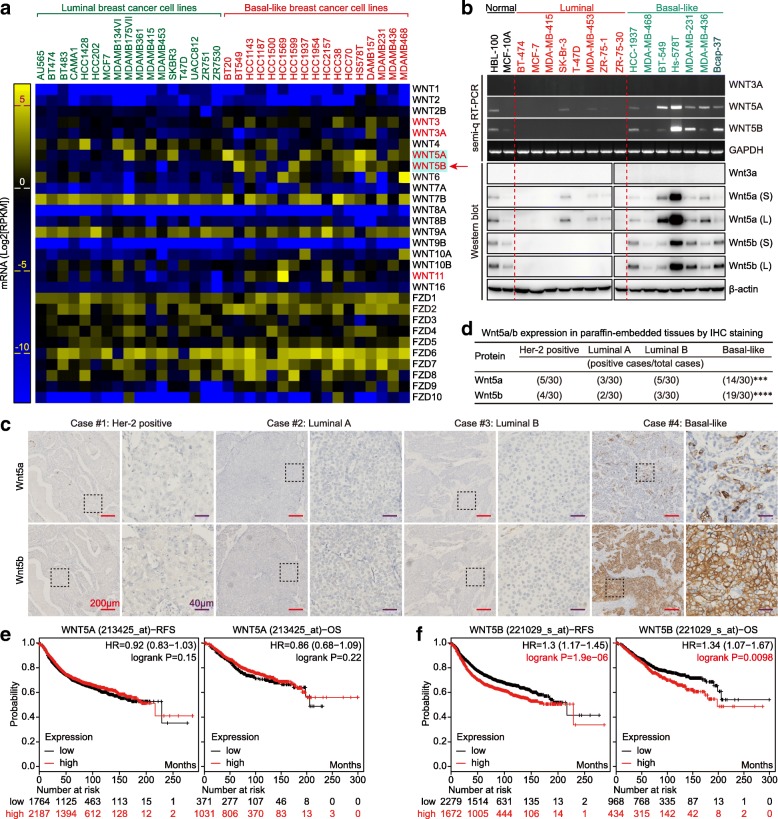
WNT5B is a specific marker of BLBC with poor prognosis. **a** mRNA expression of WNT ligands and their receptors Frizzleds between luminal and BLBC cell lines are shown in the heatmap (data are extracted from CCLE and displayed by using GraphPad Prism 7 software). **b** mRNA and protein expression levels of WNT3A, WNT5A, and WNT5B in two normal breast cell lines, eight luminal, and seven BLBC cell lines by semi-q RT-PCR and western blot (S: Short exposure; L: Long exposure). **c** Expression of Wnt5a and Wnt5b in representative Her-2 positive, luminal A, luminal B and BLBC tissues by IHC (Red scale bar = 200 μm; Purple scale bar = 40 μm). **d** The association of Wnt5a or Wnt5b expression between basal-like and non-BLBC tissue subtypes were assessed using Pearson’s χ^2^ test (***p = 0.0003, ****p < 0.0001). Prognostic value of WNT5A (**e**) and WNT5B (**f**) mRNA levels in human breast cancer, data obtained from the KM-plotter

To explain why the Wnt targets (especially Met, CD44, and TCF1/7) are preferentially expressed in BLBC. We first selected 34 breast cancer cell lines with known molecular typing [22], and analyzed the expression of Wnt ligands and their receptors based on CCLE. We found that WNT3, WNT5A, WNT5B and WNT11 were preferentially overexpressed in BLBC cell lines at mRNA level (Fig. 4a). In view of (Fig. 3a), we analyzed the expression of WNT3A, WNT3, WNT5A, WNT5B and WNT11 in bc-GenExMiner and UALCAN, and found that WNT5B was preferentially overexpressed in both BLBC and TNBC at mRNA level, while WNT11 was preferentially overexpressed in BLBC but not TNBC (Additional file 1: Figure S2b & c). Next, we analyzed the OS and RFS between WNT3/11-low and WNT3/11-high group by using KM-plotter. WNT3-high group showed good prognosis of OS and RFS (Additional file 1: Figure S2d), while WNT11 exhibited good prognosis of OS but not RFS (Additional file 1: Figure S2e). Therefore, WNT3 and WNT11 were not the key factors for BLBC phenotype shaping, while WNT5B exhibited BLBC and TNBC-specificity. WNT3A is the most well-known canonical Wnt signaling ligand, and was used as a control ligand in the following study. WNT5A has been proved as an important factor for maintaining the cancer stem cell (CSC)-like, tumorigenicity and BLBC phenotype in BLBC cells, was also used in the following study [21].

We Next analyzed the expression of WNT3A, WNT5A, and WNT5B across various normal tissues based on GTEx, and found that WNT3A was not expressed in normal breast tissues, while WNT5A and WNT5B were expressed in normal breast tissues (Additional file 1: Figure S2f). We further examined the expression of WNT3A, WNT5A, and WNT5B in the indicated cell lines at both the mRNA and protein levels, and found that WNT3A was also not expressed in in breast cancer cell lines, while WNT5A and WNT5B were preferentially expressed in BLBC cell lines (Fig. 4b) (note: normal breast cell lines HBL-100 and MCF-10A also belong to the basal-like type [22]). Moreover, Wnt5a and Wnt5b were preferentially expressed in BLBC clinical samples (Fig. 4c and d). Higher expression of WNT5B, but not WNT5A correlated with poorer prognosis (Fig. 4e and f). Thus, WNT5B is a potential biomarker of BLBC.

Besides, we examined the expression of Wnt constitutive components, including LDL receptor-related protein 6 (LRP6), disheveled segment polarity protein 2 (DVL2), DVL3, APC, Axin1, Casein kinase 1α (CK1α), GSK-3β, active β-Catenin (Np-Ser45, Np-Ser33/37/Thr41, p-Ser552, p-Ser675), inactive β-Catenin (p-Thr41/Ser45, p-Ser33/37/Thr41), and total β-Catenin in the indicated cell lines. We found that CK1α was specifically low expressed in BLBC cell lines. However, β-Catenin expression was not significantly different between the luminal group and basal-like group (Additional file 1: Figure S3a). Meanwhile, LRP6 and DVL3 were preferentially overexpressed, while CSNK1A1 (encoding CK1α) was preferentially low-expressed in BLBC or TNBC at the mRNA level (Additional file 1: Figure S3b & c). Total β-Catenin and active β-Catenin (Np-Ser45, Np-Ser33/37/Thr41) expressions were also not obviously different between Her-2+, luminal A, luminal B, and BLBC tissues (Additional file 1: Figure S3d). Similarly, β-Catenin was located in cell membrane in both luminal and basal-like cancer cell lines (Additional file 1: Figure S3e). These results suggested that canonical Wnt/β-Catenin is not the crucial signaling pathway for BLBC phenotype shaping.

### WNT5B is positively correlated with basal-like phenotype, and inversely correlated with luminal phenotype

**Fig. 5:**
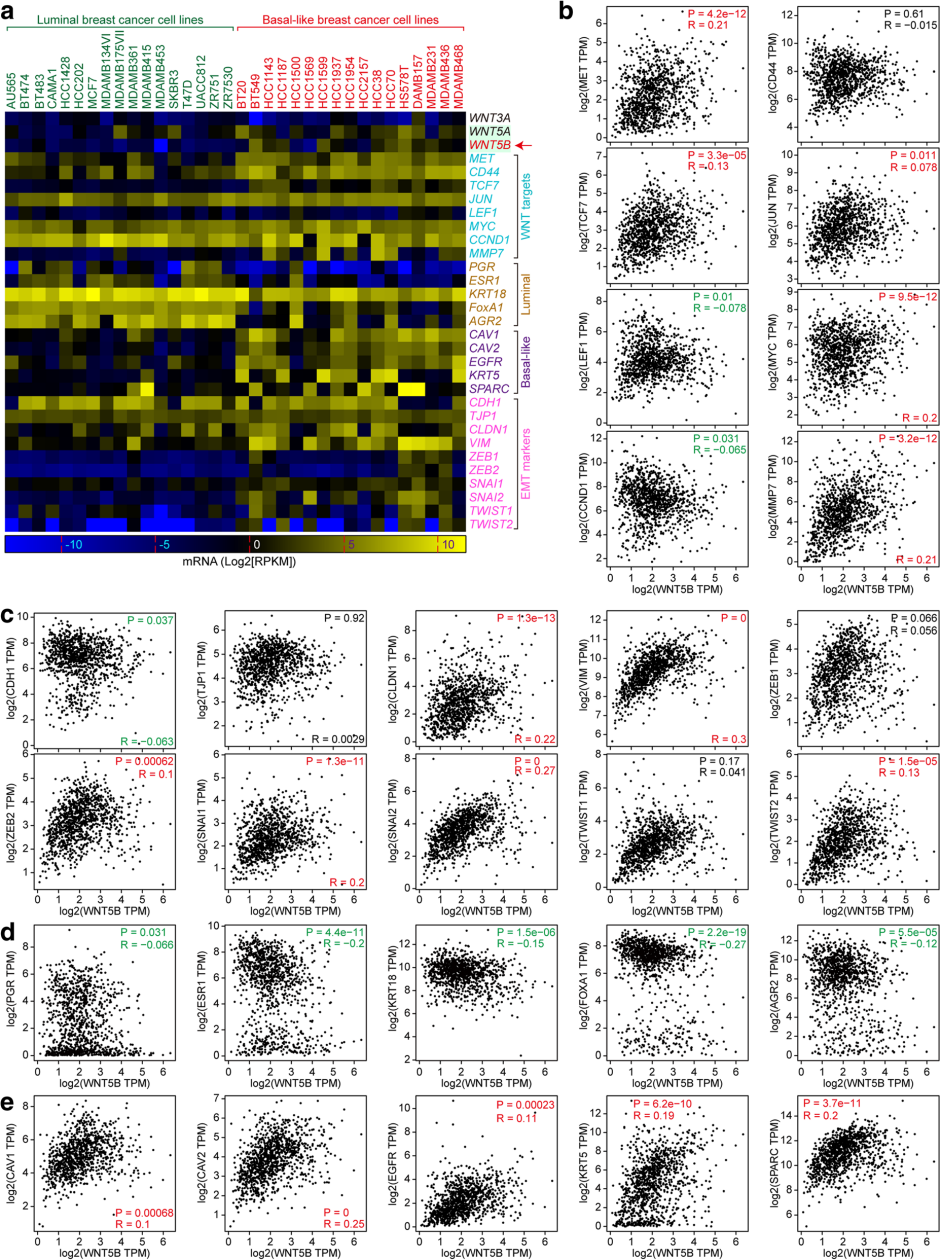
WNT5B is positively correlated with WNT signaling targets, EMT and basal-like markers, but inversely correlated with luminal markers. **a** WNT3A, WNT5A, WNT5B, WNT signaling targets, EMT markers, basal-like markers, and luminal markers mRNA expression between luminal and BLBC cell lines are shown in the heatmap (data are extracted from CCLE and displayed by using GraphPad Prism 7 software). **b** WNT5B is positively correlated with most of WNT signaling targets. **c** WNT5B is positively correlated with most of EMT markers. **d** WNT5B is inversely correlated with luminal markers. **e** WNT5B is positively correlated with basal-like markers. **b**-**e** are based on GEPIA (breast invasive carcinoma; *n* = 1085; Red: positive correlation; Green: negative correlation; Black: no correlation)

To explore the correlation between WNT5B and basal-like phenotype, we selected 34 breast cancer cell lines with known molecular typing [22] based on CCLE. As expected, WNT5B, Wnt targets (MET, CD44, TCF7, and MMP7), acquired EMT markers, and basal-like markers were preferentially expressed in BLBC cell lines (Fig. 5a). Then we analyzed the correlation between WNT5B and Wnt targets, EMT, luminal, and basal-like markers, and found that WNT5B was positively correlated with most Wnt targets, except LEF1 and CCND1 (CCND1 showed luminal-specificity) (Fig. 5b). Besides, WNT5B was positively correlated with most acquired EMT markers but was inversely correlated with attenuated EMT marker (CDH1) (Fig. 5c). Notably, WNT5B was inversely correlated with all luminal markers (Fig. 5d) but positively correlated with all basal-like markers (Fig. 5e). Thus, WNT5B is a potential factor for shaping the phenotype of BLBC cells.

### WNT5B governs the phenotype of BLBC by activating canonical and non-canonical Wnt signaling

**Fig. 6:**
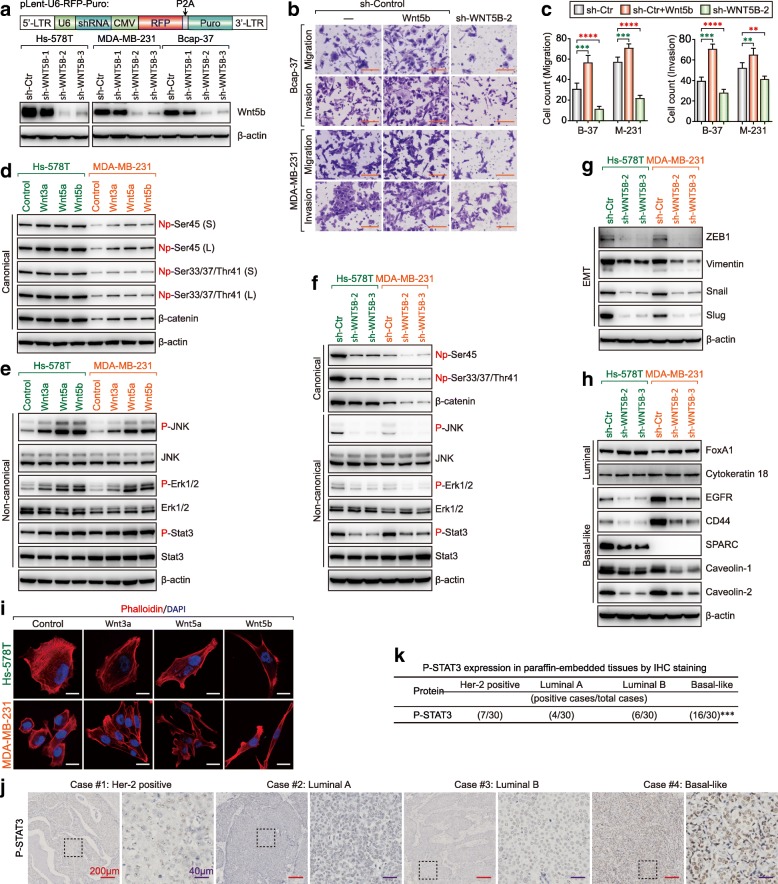
WNT5B controls the phenotype of BLBC by activating canonical and non-canonical WNT signaling. **a** Western blot showing Wnt5b upon knockdown of WNT5B by lentivirus-mediated shRNA in Hs-578 T, MDA-MB-231, and Bcap-37 cells. **b** Migration and invasion of sh-control with or without Wnt5b ligand and Wnt5b-KD Bcap-37 cells and MDA-MB-231 cells were measured using transwell chamber assays (scale bar = 100 μm). **c** Data represent the average cell number from 5 viewing fields (B-37: Bcap-37; M-231: MDA-MB-231; **p < 0.005, ***p < 0.001, ****p < 0.0001). **d** Active β-Catenin (Non-phospho-ser45 and Non-phospho-Ser33/37/Thr41 and total β-Catenin were analyzed by western blot (Hs-578 T and MDA-MB-231 cells were pretreated with Wnt3a, Wnt5a, or Wnt5b; S: Short exposure; L: Long exposure). **e** Protein level of phospho-JNK (Thr183/Tyr185), JNK, phospho-Erk1/2 (Thr202/Tyr204), Erk1/2, phospho-STAT3 (Tyr705), and STAT3 were analyzed by western blot (Hs-578 T and MDA-MB-231 cells were pretreated with Wnt3a, Wnt5a, or Wnt5b). **f** Protein level of canonical and non-canonical markers in WNT5B-KD Hs-578 T and MDA-MB-231 cells was analyzed by western blot. **g** Protein level of EMT markers in WNT5B-KD Hs-578 T and MDA-MB-231 cells was analyzed by western blot. **h** Protein level of luminal and basal-like markers in WNT5B-KD Hs-578 T and MDA-MB-231 cells was analyzed by western blot. **i** Cytoskeleton F-actin proteins were stained with phalloidin and viewed under a confocal microscope (scale bar = 20 μm). **j** Protein expression of phospho-STAT3 (Tyr705) in representative Her-2 positive, luminal A, luminal B and BLBC tissues by IHC (Red scale bar = 200 μm; Purple scale bar = 40 μm). **k** The association of phospho-STAT3 (Tyr705) expression between basal-like and non-BLBC tissue subtypes were assessed using Pearson’s χ^2^ test (***p = 0.0003)

Typical BLBC cell lines: Hs-578 T, MDA-MB-231 and newly identified Bcap-37 were chosen for subsequent studies. Lentivirus-mediated shRNA was used to knockdown Wnt5b expression in Hs-578 T, MDA-MB-231, and Bcap-37 cells. sh-WNT5B-2 and WNT5B-3 showed high efficiency of knockdown and were chosen for further studies (Fig. 6a). Recombinant Wnt5b promoted the migration and invasion of Bcap-37 and MDA-MB-231 cells, while the knockdown of endogenous Wnt5b inhibited migration and invasion of Bcap-37 and MDA-MB-231 cells. These suggested that Wnt5b is an important factor for BLBC cell migration and invasion (Fig. 6b and c). Recombinant Wnt5b upregulated active and total β-Catenin slightly similar to Wnt3a and Wnt5a (Fig. 6d); in addition, it also activated non-canonical Wnt signaling by promoting phosphorylation of JNK, Erk1/2, and Stat3 significantly as with Wnt5a (Fig. 6e). Active and total β-Catenin was reduced in Wnt5b-KD cells, and the phosphorylation of JNK, Erk1/2, and Stat3 were reduced in Wnt5b-KD cells (Fig. 6f). Besides, luminal markers FoxA1 and CK18 were upregulated while basal-like markers EGFR, CD44, SPARC, Caveolin-1, and Caveolin-2 were reduced in Wnt5b-KD cells (Fig. 6g). Acquired EMT markers ZEB1, vimentin, Snail, and Slug were also reduced in Wnt5b-KD cells (Fig. 6h). Recombinant Wnt5b altered the cells to exhibit a more mesenchymal-type morphology similar to Wnt5a (Fig. 6i). Stat3 was persistently phosphorylated in BLBC cells [38], so we next examined the expression of p-Stat3 in clinical samples, and found that p-Stat3 was preferentially expressed in BLBC as expected (Fig. 6j and k). These evidences suggested that WNT5B governs the phenotype of BLBC by activating both canonical and non-canonical Wnt signaling, and non-canonical Wnt is the dominant signaling pathway for BLBC phenotype shaping.

### Knockdown of WNT5B inhibits the tumorigenicity of basal-like cancer cells in vivo

**Fig. 7:**
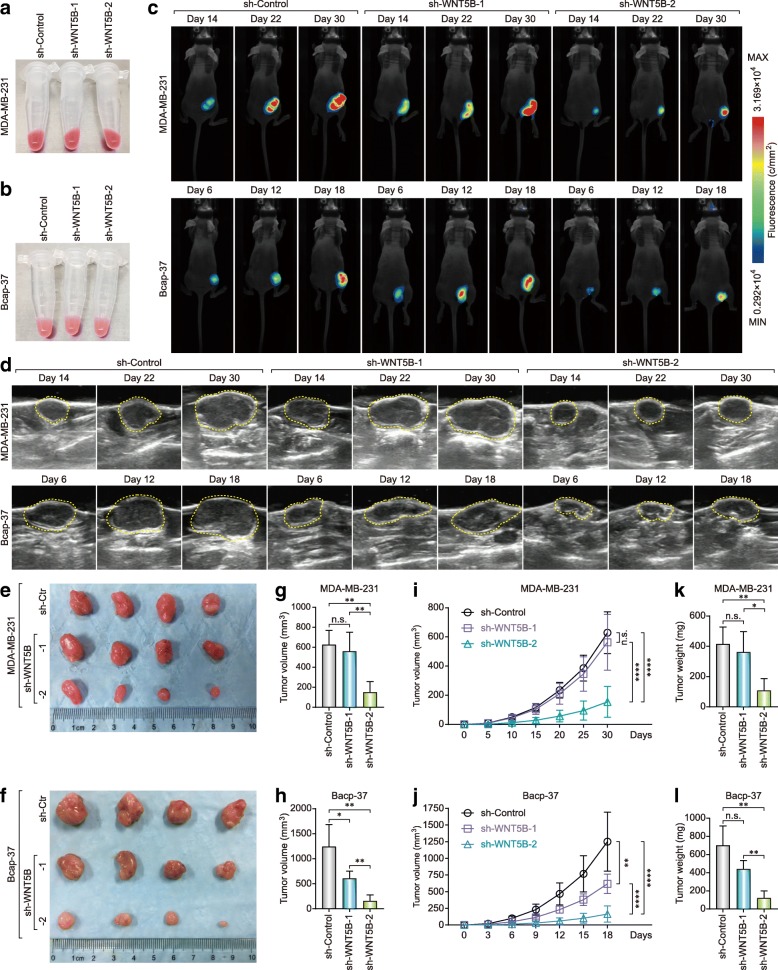
Knockdown of WNT5B inhibits the tumorigenicity of basal-like cancer cells in vivo. RFP-expressed MDA-MB-231 (**a**) and Bcap-37 (**b**) cell pellets under visible light. **c** Fluorescence images of BLBC cells bearing mice under 530 nm laser irradiation at day 14, 22, and 30 for MDA-MB-231 or at day 6, 12, and 18 for Bcap-37 with/without WNT5B-KD. **d** Ultrasound images of BLBC cells bearing mice under B-type ultrasonic image system at day 14, 22, and 30 for MDA-MB-231 or at day 6, 12, and 18 for Bcap-37 with/without WNT5B-KD. Subcutaneous tumor of MDA-MB-231 (**e**) and Bcap-37 (**f**) with/without WNT5B-KD at the experimental endpoint. Tumor volume of MDA-MB-231 (**g**) and Bcap-37 (**h**) at the experimental endpoint (*p < 0.05; **p < 0.005). Tumor growth curve of MDA-MB-231 (**i**) and Bcap-37 (**j**) (**p < 0.005; ****p < 0.0001). Tumor weight of MDA-MB-231 (**k**) and Bcap-37 (**l**) at the experimental endpoint (*p < 0.05; **p < 0.005)

Lentivirus-mediated shRNA also expressed red fluorescent protein (RFP) (Fig. 6a), and the cells that were infected with the lentivirus showed red fluorescence under visible light (Fig. 7a and b). We used RFP to monitor tumor growth, and found that knockdown of Wnt5b inhibited the tumor growth compared to the control (Fig. 7c). Ultrasound imaging further confirmed that the tumor growth after knockdown of WNT5B was significantly inhibited (Fig. 7d). In addition, invalid RNA interference (sh-WNT5B-1) in MDA-MB-231 cells did not inhibit tumor growth, and it was also observed in Bcap-37 cells. The inhibition efficiency of tumor growth is proportional to the efficiency of RNA interference (Fig. 7e-l). These evidences suggested that Wnt5b is not only a diagnostic biomarker but also a potential therapeutic target of BLBC.

### Wnt5b is a potential therapeutic target of BLBC

**Fig. 8:**
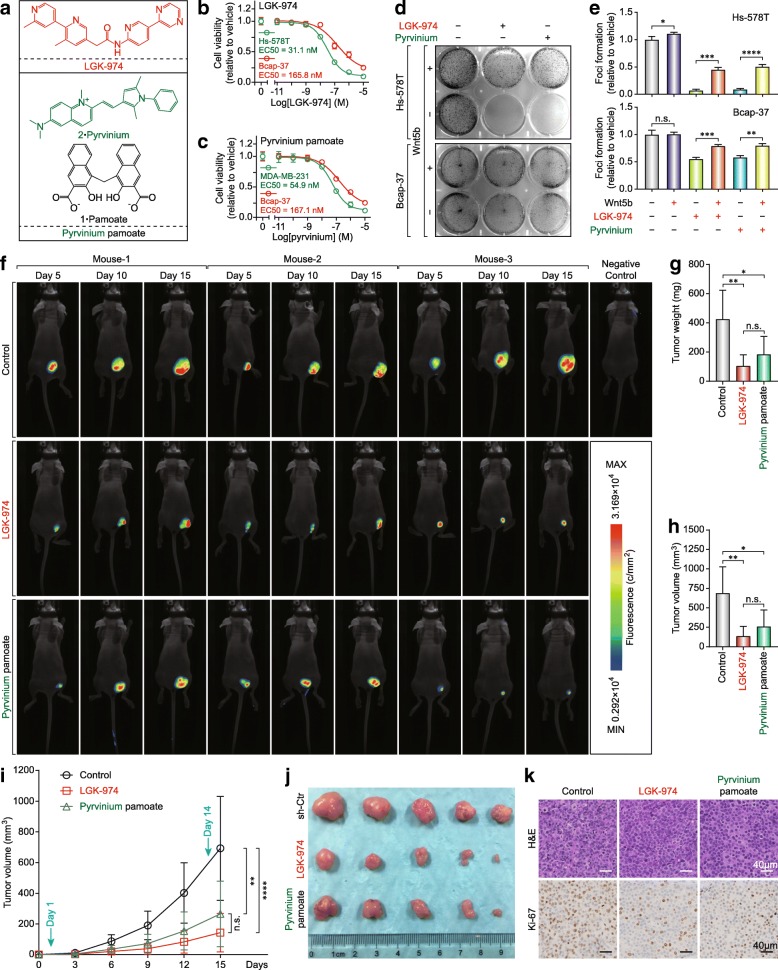
Small-molecule compounds inhibit tumor growth by targeting Wnt5b-mediated non-canonical WNT signaling and CK1α-mediated canonical WNT signaling in vitro and in vivo. **a** Chemical structure of LGK-974 and pyrvinium pamoate. Cell viability was determined in MDA-MB-231 and Bcap-37 cells following treatment with LGK-974 (**b**) and pyrvinium pamoate (**c**) for 72 h, mean ± SEM is shown (assays performed in quadruplicate). **d** LGK-974 and pyrvinium pamoate inhibited cell lines with a high expression of Wnt5b and a low expression of CK1α by foci formation assay. **e** Graphs represent LGK-974-treated or pyrvinium pamoate-treated Hs-578 T and Bcap-37 with or without Wnt5b (500 ng/ml), mean ± SD (*n* = 3 per treatment group), (n.s.: no significant, *p < 0.05, **p < 0.005, ***p < 0.001, ****p < 0.0001). **f** Images of Bcap-37 cells bearing mice under 530 nm laser irradiation at day 5, 10, and 15 following treatment with LGK-974 (5 mg/kg) or pyrvinium pamoate (1 mg/kg). Tumor weight (**g**), Tumor volume (**h**), Tumor growth curve (**i**), and subcutaneous tumor (**j**) of Bcap-37 at the experimental endpoint. **k** Representative images of Ki-67 staining by IHC after 14 days of treatment

Wnt undergoes post-translational acylation (palmitoylation) mediated by Porcupine, a membrane-bound O-acyltransferase [39] (Additional file 1: Figure S4a). LGK-974 (also known as WNT-974, a Phase I drug for TNBC and other Wnt ligands-dependent cancers) is a Porcupine inhibitor and can inhibit Wnt5a and Wnt5b secretion effectively [40]. On the other hand, CK1α, a canonical Wnt signaling inhibitor showed low-expression in BLBC (Additional file 1: Figure S3a), and was positively correlated with luminal markers and some attenuated EMT markers and was inversely correlated with some basal-like, acquired EMT markers and WNT5B (Additional file 1: Figure S4b-g). pyrvinium is a potent inhibitor of Wnt signaling which acts by activating CK1α [41]. Therefore, LGK-974 and pyrvinium were selected for further studies (Fig. 8a). Hs-578 T with HRAS mutant (G12D) and Wnt5a^high^/ Wnt5b^high^ was more sensitive to LGK-974 than Bcap-37 (Fig. 8b) and MDA-MB-231 with KRAS mutant (G13D) was more sensitive to pyrvinium than Bcap-37 (Fig. 8c). In addition, the foci formation assay showed that Hs-578 T was more sensitive to LGK-974 and pyrvinium than Bcap-37, and Wnt5b reversed the foci formation partly (Fig. 8d and e). Moreover, LGK-974 and pyrvinium also inhibited the tumor growth in Bcap-37 cells bearing mice xenograft model (Fig. 8f-j and Additional file 1: Figure S4h).

The reduction of the proliferation marker Ki-67 was also observed in the xenograft tumors (Fig. 8k and Additional file 1: Figure S4i). In summary, Wnt5b governs the phenotype of BLBC by activating both canonical and non-canonical Wnt signaling (Additional file 1: Figure S5), and should be a promising theranostic target of basal-like breast cancer.

## Discussion

BLBC is associated with aggressive behavior, stem-like phenotype, high histological grade, poor clinical features, and high rates of recurrences and/or metastasis [42]. However, the mechanisms shaping the phenotype of BLBC remain obscure. In this study, we provide several mechanistic insights on how Wnt5b governs the phenotype of BLBC.

Wnt signaling activation has been observed in breast, lung, and hematopoietic malignancies and contributes to tumor recurrence [43], and is a promising therapeutic target for breast cancer therapy [44]. However, the relationship between Wnt signaling and breast cancer is still largely unknown, especially with regard to the molecular type of breast cancer. We analyzed the sequenced invasive breast carcinoma cases/patients from cBioPortal and found that *Myc* and *CCND1* were highly amplified at the DNA level in breast cancer. We further found that most Wnt targets were preferentially expressed in BLBC, except Cyclin D1, which exhibited luminal-specificity. Then, we analyzed the canonical Wnt signaling constitutive components, but no significant differences were found between basal-like and luminal breast cancers, especially the various forms of β-Catenin, although CK1α expressed at low levels was identified in BLBC. Surprisingly, the unchanged β-Catenin induced activation of Wnt targets in BLBC. Thus, canonical Wnt signaling is not the leading factor for basal-like phenotype shaping, and there must be another mechanism.

Previous studies indicated that non-canonical Wnt ligands Wnt5a or/and Wnt5b were preferentially expressed in BLBC [11, 21, 45, 46], or TNBC [47]. Our results were in line with these previous reports and we found that Wnt5a and Wnt5b were preferentially expressed in BLBC; in addition, Wnt5b was superior to Wnt5a for molecular typing, as well as in prognosis estimation. BLBC has a high incidence of metastasis and shows high expression of acquired EMT markers [9, 21]. Wnt5a and Wnt5b have been proven as regulators of EMT in breast cancer [21, 45], and we also corroborated this conclusion. Moreover, we found that Wnt5b is positively correlated with basal-like phenotype, but inversely correlated with luminal phenotype for the first time, and also demonstrated that Wnt5b governs the phenotype of BLBC by activating canonical and non-canonical Wnt signaling. These results are consistent with the findings of previous studies [21, 48, 49].

A recent study demonstrated that Wnt5b promotes cancer cell migration and proliferation by exosome-mediated secretion. Knockdown of Wnt5b decreased the exosome-mediated secretion and inhibited Wnt5b-dependent cell proliferation and migration [49]. Therefore, Wnt5b may be a promising target for BLBC treatment. We further knocked down Wnt5b in MDA-MB-231 and Bcap-37 cells, which significantly inhibited the tumor growth.

The lentivirus transfected cells also expressed RFP and the quantification of the red fluorescence using external fluorescence imaging correlated strongly with tumor volume as reported previously [50, 51]. Thus, RFP is useful for superficial tumor growth monitoring for cancer cells with Wnt5b-KD or treated with small molecules.

Surprisingly, CK1α, a Wnt signaling inhibitor [52], was expressed at a higher level in luminal cancers than in BLBC cell lines. Therefore, Wnt5b and CK1α were selected as therapeutic targets. LGK-974, a Porcupine inhibitor inhibited Wnt5a and Wnt5b secretion effectively [40], and was chosen for Wnt5b targeted treatment, while pyrvinium, an activator [41] of CK1α was also chosen for CK1α targeted treatment, although the various phosphorylated states (Ser-45) of β-Catenin showed no significant difference between luminal and BLBC cell lines. Hs-578 T with HRAS mutant (G12D) and Wnt5a^high^/ Wnt5b^high^ was more sensitive to LGK-974 than Bcap-37, and exogenous Wnt5b attenuated the effect of LGK-974 to a certain extent as expected. The results further strengthen the evidence for the clinical utility of LGK-974.

MDA-MB-231 with KRAS mutant (G13D) was more sensitive to pyrvinium than Bcap-37, but total or active β-Catenin was expressed in MDA-MB-231 and Bcap-37 at a very low level. In fact, CK1α is a multifunctional protein involved in various signaling pathways [52] including Wnt/β-catenin, Hedgehog, autophagy, NF-κB, etc. Activation of CK1α by pyrvinium also inhibited the proliferation and tumor growth via attenuation of the Hedgehog signaling pathway [53] or inhibition of autophagosome formation [54]. Previous studies reported that aggressive breast cancer cell lines SUM-149 and SUM-159 (both belong to the basal-like type [22]) were inhibited in vitro and in vivo [55]. However, RAS mutant cells seem more sensitive to LGK-974 and pyrvinium. Bcap-37 xenograft tumors treated with LGK-974 and pyrvinium reduced the expression of proliferation marker Ki-67 as reported previously [35, 56].

Nevertheless, there are still some limitations of this study. Firstly, Cyclin D1, a common Wnt target is preferentially expressed in luminal breast cancer, which is inconsistent with other Wnt targets, suggesting that Cyclin D1 amplification may occur in luminal subtype preferentially. Secondly, no or very few *CTNNB1* mutations or CNA (especially the amplification and deletion) were observed in breast cancer, as well as the cell lines used in this study. Although CK1α is expressed at low levels in BLBC, no significant differences in total or active β-Catenin (Ser-45) expression were found between basal-like and luminal breast cancers. Thirdly, Wnt5b exhibited basal-like specificity in cells and clinical samples at both the mRNA and protein levels, and also showed good correlation with basal-like phenotype at the mRNA level, but about one third the cases were Wnt5b negative, suggesting that Wnt5b may collaborate with Wnt5a (or other factors) to maintain the phenotype of BLBC. Insights into the essential characteristics of BLBC should promote a better understanding of its phenotype and open new avenues for its diagnosis and treatment.

## Conclusions

In this study, we evaluated various factors contributing to the physical and physiological phenotype of BLBC and identified Wnt5b as a key regulatory factor that governs the phenotype of BLBC by activating canonical and non-canonical Wnt signaling. Wnt5b exhibited basal-like specificity in cells and clinical samples at both the mRNA and protein levels, and also showed good correlation with basal-like phenotype at the mRNA level. Besides, Wnt5b is a promising therapeutic target for LGK-974 treatment. It will provide a new insight into the diagnosis and treatment of BLBC. In addition, we identified that CK1α was expressed at low levels in BLBC and that the activation of CK1α by pyrvinium was an alternative strategy for BLBC treatment.

## Additional file

Additional file 1: **Figure S1.** Expression of canonical breast cancer subtype-markers in breast cancer. **a** Expression of selected canonical breast cancer subtype-marker mRNAs with Her-2 positive-specific, luminal-specific, or TNBC-specific based on UALCAN (Red p: the former > the latter; Green p: the former < the latter). **b** Common breast cancer subtype-marker expression in representative Her-2 positive (Her-2+), luminal A (ER/PR+, Her-2-, EGFR-, Ki-67% < 14%), luminal B (ER/PR+, Her-2-, EGFR-, Ki-67% ≥ 15%), and BLBC (ER-, Her-2-, EGFR+, CK5/6+) tissues by IHC (Red scale bar = 200 μm; Purple scale bar = 40 μm). **Figure S2.** Identification of BLBC-specific Wnt ligands based on online platforms. **a** mRNA expression of selected canonical WNT signaling targets with TNBC or non-luminal-specific based on UALCAN (Red p: the former > the latter; Green p: the former < the latter). **b** mRNA expression of WNT3A, WNT3, WNT5A, WNT5B, and WNT11 in five different breast cancer subtypes based on bc-GenExMiner v4.1 according to the Sørlie’s subtypes (****p < 0.0001; Red star: the former > the latter; Green Star: the former < the latter). **c** mRNA expression of WNT3A, WNT3, WNT5A, WNT5B, and WNT11 in normal breast, Luminal, Her-2 positive, and TNBC subtypes based on UALCAN (Red p: the former > the latter; Green p: the former < the latter). Prognostic value of WNT3 **d** and WNT11 **e** mRNA levels in human breast cancer, data obtained from the KM-plotter. **f** WNT3A, WNT5A, and WNT5B mRNA expression levels across various normal tissues based on GTEx which were deposited in the HPA. **Figure S3.** Analysis of canonical WNT signaling constitutive components in breast cancer. **a** Expression levels of canonical WNT signaling constitutive components in two normal breast cell lines, eight luminal, and seven BLBC cell lines by western blot (S: Short exposure; L: Long exposure). **b** Expression of selected canonical WNT signaling constitutive component mRNAs with luminal or basal-like specific based on bc-GenExMiner v4.1 according to the Sørlie’s subtypes (*p < 0.05; ****p < 0.0001; Red star: the former > the latter; Green Star: the former < the latter). **c** Expression of selected canonical WNT signaling constitutive component mRNAs with TNBC or non-TNBC-specific based on UALCAN (Red p: the former > the latter; Green p: the former < the latter). **d** Expression of β-catenin and active β-catenin (np-Ser45, np-Ser33/37/Thr41) in representative Her-2 positive, luminal A, luminal B and BLBC tissues by IHC (Red scale bar = 200 μm; Purple scale bar = 40 μm). **e** Expression of β-catenin in two luminal and two BLBC lines by immunofluorescence (confocal microscopy, scale bar = 20 μm). **Figure S4.** Identifying inhibitors targeting Wnt5b and CK1α based on online platforms. **a** PPI pattern of WNT3A, WNT5A, and WNT5B based on STRING. **b** CSNK1A1 is positively correlated with luminal markers. **c** CSNK1A1 is inversely correlated with basal-like marker KRT5. **d** CSNK1A1 is positively correlated with EMT-attenuated markers CDH1 and TJP1. **e** CSNK1A1 is inversely correlated with EMT acquired markers VIM and SNAI1. **f** CSNK1A1 is inversely correlated with WNT5B. **g** CSNK1A1 is positively correlated with CCND1. **(b**-**g)** were based on GEPIA (breast invasive carcinoma; *n* = 1085). **h** Ultrasound images of BLBC cells bearing mice under B-type ultrasonic image system at day 6, 12, and 18 for Bcap-37 following treatment with LGK-974 (5 mg/kg) or pyrvinium pamoate (1 mg/kg). **i** Percentage of nuclei positive for Ki67 after 14 days of LGK-974 or pyrvinium pamoate treatment. Graphs represent mean ± SEM (*n* = 5 per treatment group; ****p < 0.0001, n.s.: no significant). **Figure S5.** Proposed model of WNT5B governing the phenotype of BLBC by activating canonical and non-canonical WNT signaling. (PDF 14908 kb)

## Data Availability

Not applicable.

## Abbreviations

AGR2: Anterior gradient homolog 2
APC: Adenomatous polyposis coli
BLBC: Basal-like breast cancer
CK18: Cytokeratin 18
CK1α: Casein kinase 1α
CK5: Cytokeratin 5
EGFR: Epidermal growth factor receptor
EMT: Epithelial-mesenchymal transition
ER: Estrogen receptor
FoxA1 (also known as HNF3α): Foxkhead box A1
GSK-3α/β: Glycogen synthase kinase 3α/β
Her-2: Human epidermal growth factor 2
IHC: Immunohistochemistry
LEF1: Lymphoid Enhancer Factor 1
LRP6: Low-density lipoprotein receptor-related protein 6
PR: Progesterone Receptor
SPARC: Secreted protein acidic and rich in cysteine
TCF1 (also known as TCF7): T Cell Factor 1
TNBC: Triple negative breast cancer
TWIST1: Twist family bHLH transcription factor 1
ZEB1 (also known as TCF8): Zinc finger E-box-binding homeobox 1
ZO-1: Zona occludens-1
