# Supplementary figures and images for: WNT5B governs the phenotype of basal-like breast cancer by activating WNT signaling
Source: Cell Commun Signal. 2019 Aug 28;17:109. doi: 10.1186/s12964-019-0419-2 (PMC6714433; doi:10.1186/s12964-019-0419-2)

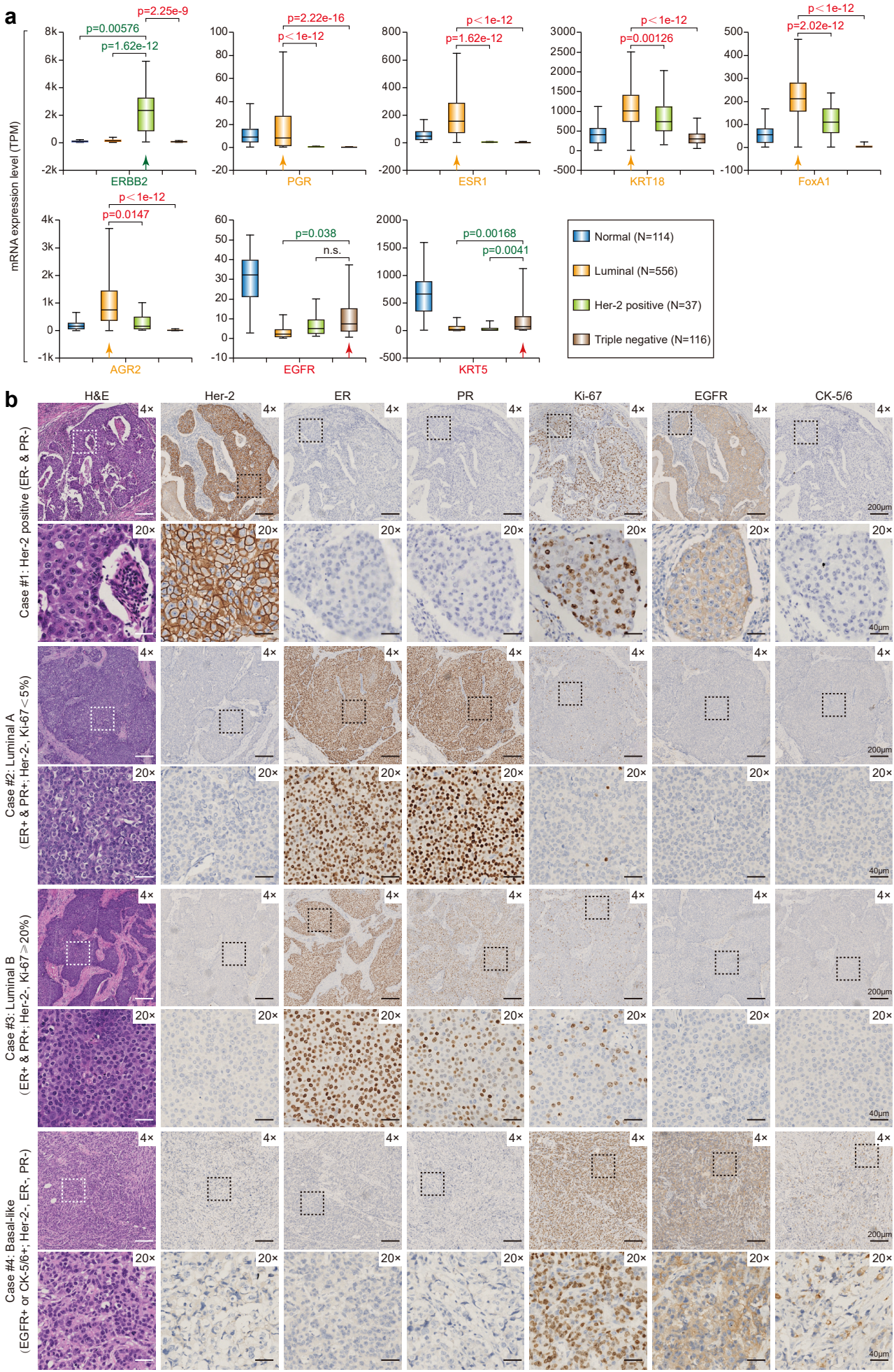

Figure-S2

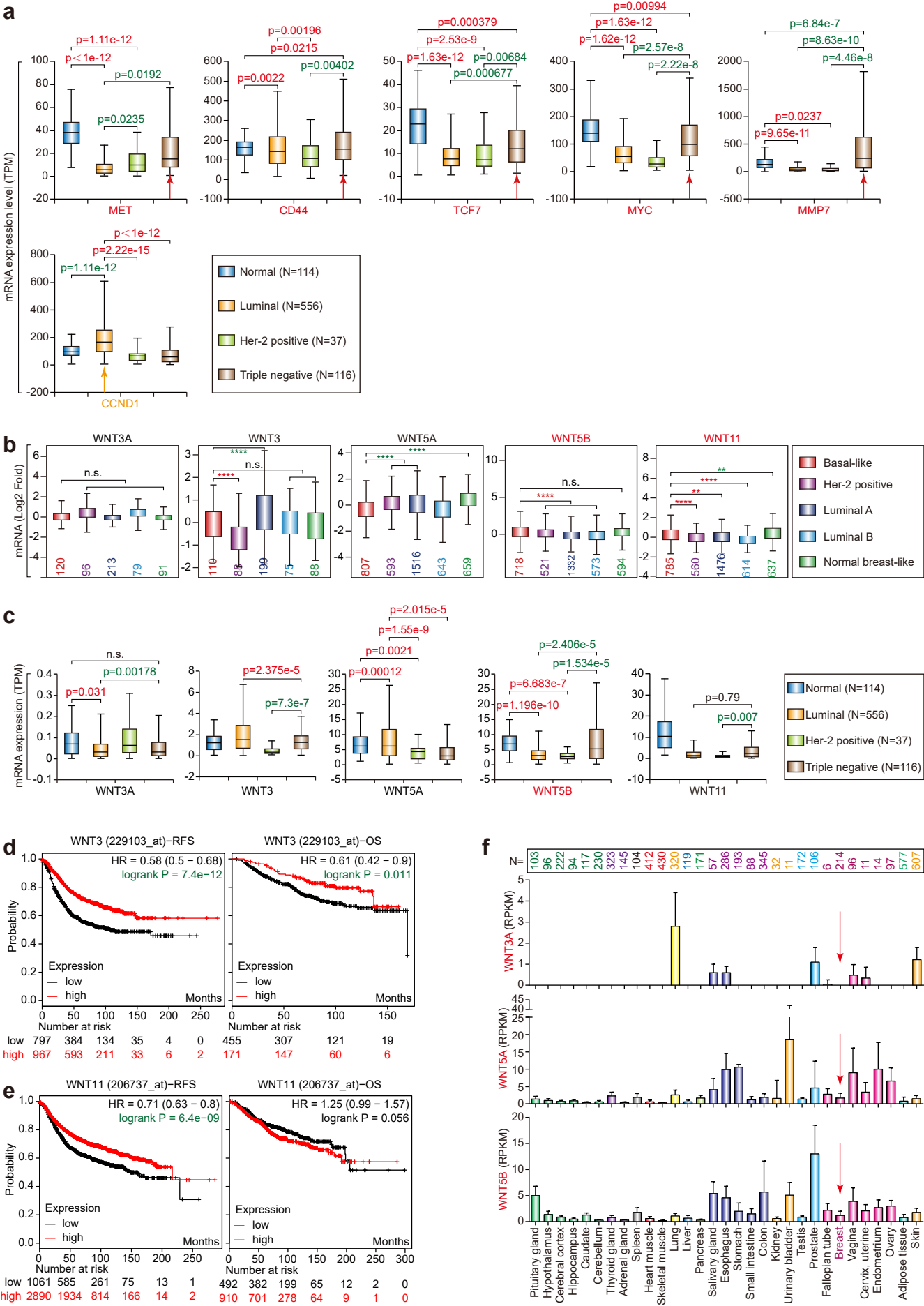

Figure-S3

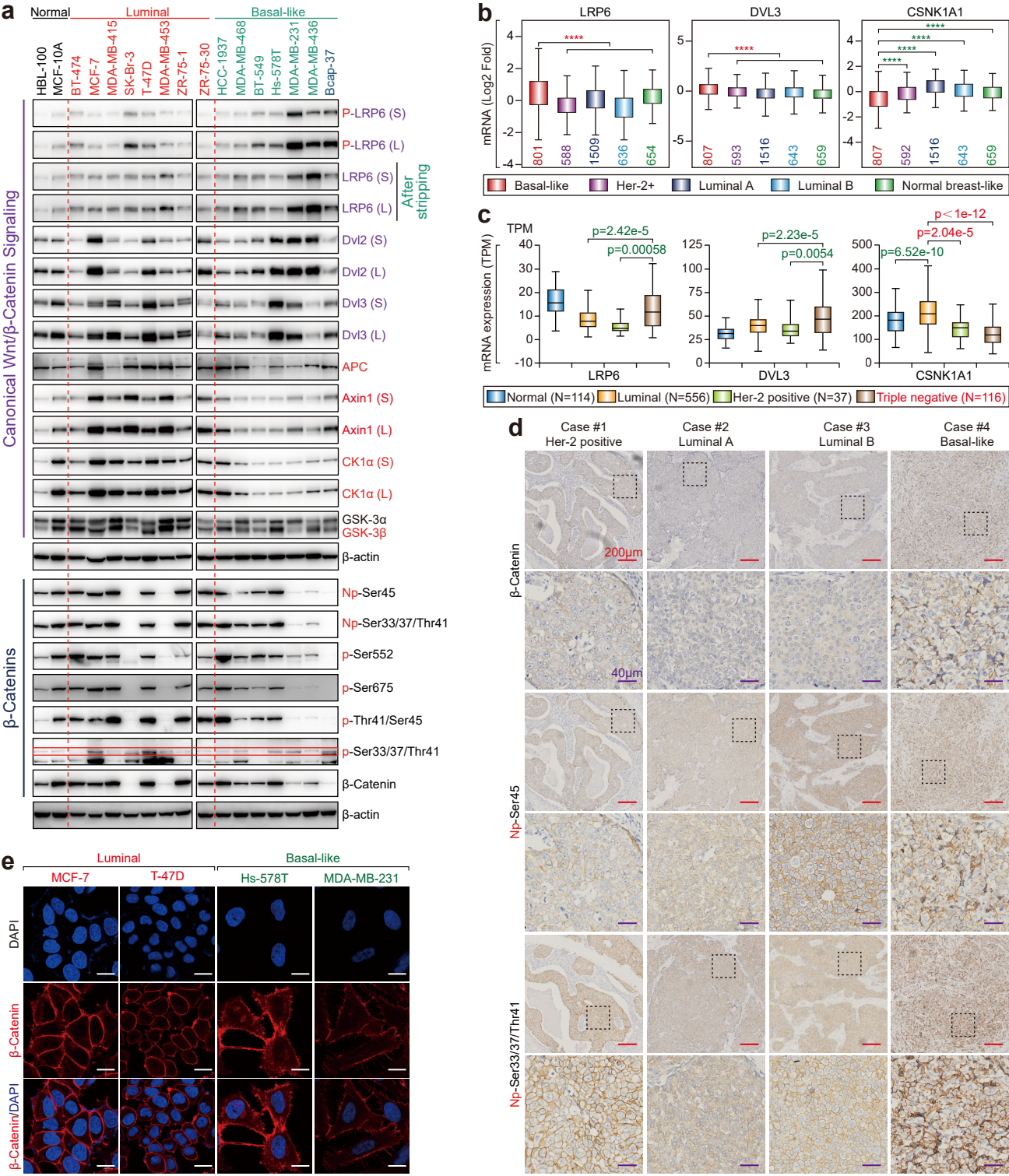

Figure-S4

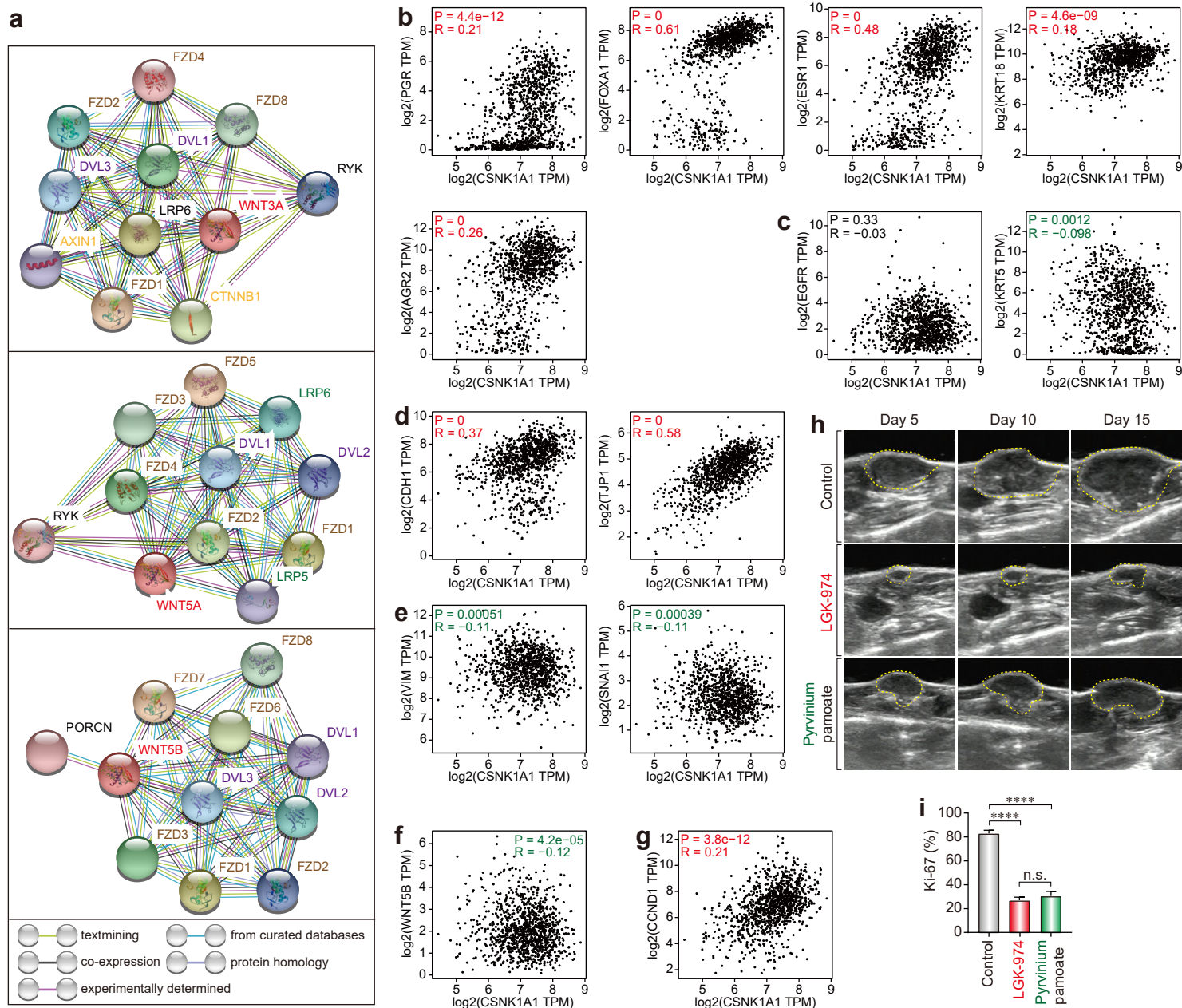

Figure-S5

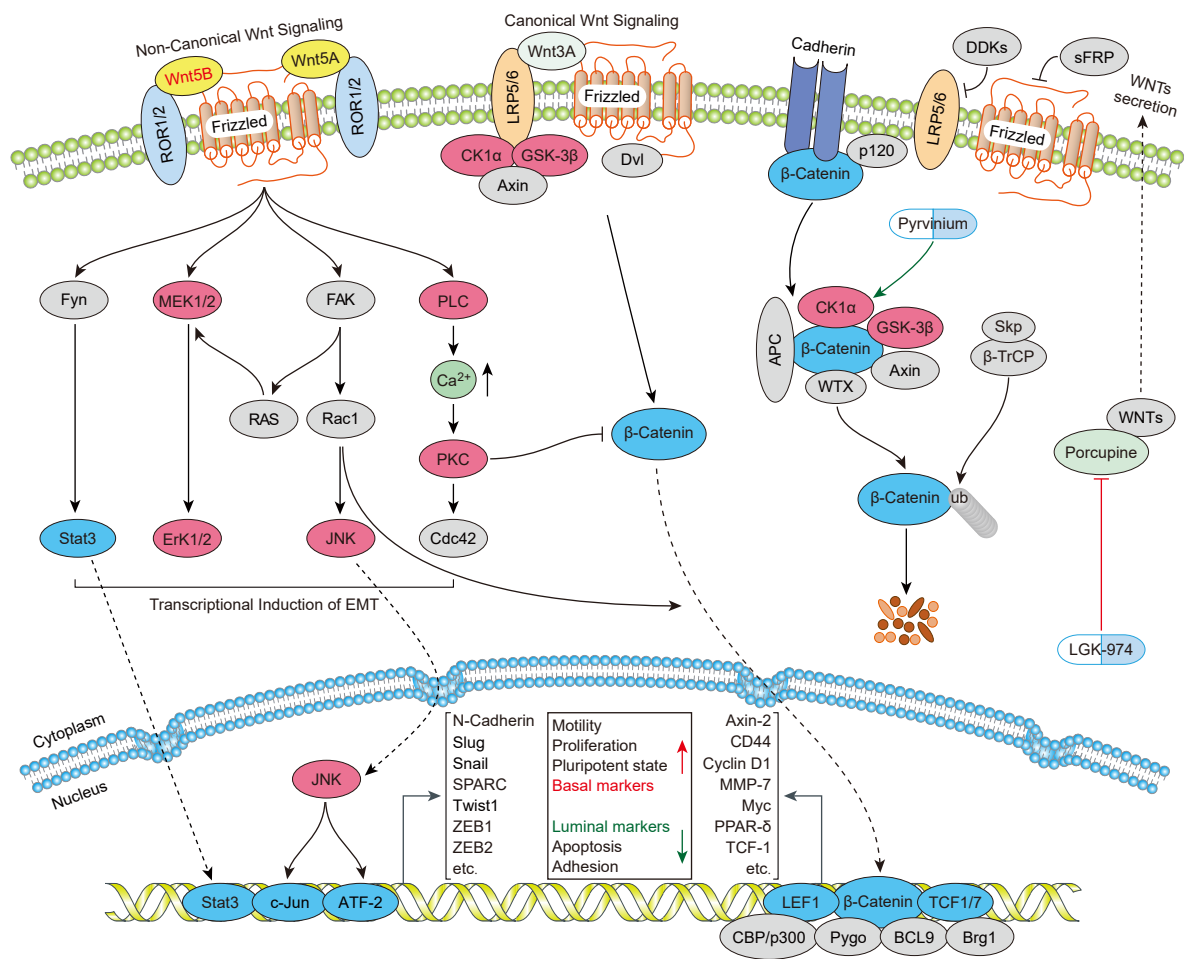

Supplement: Supplementary file 1 — Figure S1. Expression of canonical breast cancer subtype-markers in breast cancer. a Expression of selected canonical breast cancer subtype-marker mRNAs with Her-2 positive-specific, luminal-specific, or TNBC-specific based on UALCAN (Red p: the former > the latter; Green p: the former < the latter). b Common breast cancer subtype-marker expression in representative Her-2 positive (Her-2+), luminal A (ER/PR+, Her-2-, EGFR-, Ki-67% < 14%), luminal B (ER/PR+, Her-2-, EGFR-, Ki-67% ≥ 15%), and BLBC (ER-, Her-2-, EGFR+, CK5/6+) tissues by IHC (Red scale bar = 200 μm; Purple scale bar = 40 μm). Figure S2. Identification of BLBC-specific Wnt ligands based on online platforms. a mRNA expression of selected canonical WNT signaling targets with TNBC or non-luminal-specific based on UALCAN (Red p: the former > the latter; Green p: the former < the latter). b mRNA expression of WNT3A, WNT3, WNT5A, WNT5B, and WNT11 in five different breast cancer subtypes based on bc-GenExMiner v4.1 according to the Sørlie’s subtypes (****p < 0.0001; Red star: the former > the latter; Green Star: the former < the latter). c mRNA expression of WNT3A, WNT3, WNT5A, WNT5B, and WNT11 in normal breast, Luminal, Her-2 positive, and TNBC subtypes based on UALCAN (Red p: the former > the latter; Green p: the former < the latter). Prognostic value of WNT3 d and WNT11 e mRNA levels in human breast cancer, data obtained from the KM-plotter. f WNT3A, WNT5A, and WNT5B mRNA expression levels across various normal tissues based on GTEx which were deposited in the HPA. Figure S3. Analysis of canonical WNT signaling constitutive components in breast cancer. a Expression levels of canonical WNT signaling constitutive components in two normal breast cell lines, eight luminal, and seven BLBC cell lines by western blot (S: Short exposure; L: Long exposure). b Expression of selected canonical WNT signaling constitutive component mRNAs with luminal or basal-like specific based on bc-GenExMiner v4.1 according to the Sø [file 12964_2019_419_MOESM1_ESM.pdf]
